# A Ganglion Cyst on the Posterior Cruciate Ligament

**DOI:** 10.7759/cureus.27944

**Published:** 2022-08-12

**Authors:** Gaurav Singh, Sachin Avasthi, Satyam Singh

**Affiliations:** 1 Department of Orthopaedics, Dr Ram Manohar Lohia Institute of Medical Sciences, Lucknow, IND

**Keywords:** mri knee, intra articular cyst, intra articular cyst, posterior cruciate ligament, posterior cruciate ligament

## Abstract

This case report describes a 29-year-old medical student, a junior resident in orthopaedic surgery who reported intermittent right knee pain due to an increase in mileage throughout his training. The posterior cruciate ligament was diagnosed with a ganglion cyst using diagnostic imaging. The purpose of this article is to describe the clinical and diagnostic aspects of a ganglion cyst found on the posterior cruciate ligament and to raise awareness among doctors of this uncommon condition and its diagnosis.

## Introduction

Intra-articular ganglion cysts of the knee are rare and ganglion cysts arising from the anterior cruciate ligament (ACL) are rarely reported [1]. Intra-articular cysts were seen mostly during MRI and arthroscopy, with prevalence rates of 0.2% to 1.3% and 0.6%, respectively [2,3]. The anterior cruciate ligament is more likely than the posterior cruciate ligament (PCL) to be associated with ganglion cysts. Krudwig et al., over a 15-year period, examined 8,000 arthroscopically knees and found 85 intra-articular ganglions [4]. There were 49 ganglions in the ACL and 16 in the PCL, with the remaining ganglions in the medial plica, menisci, and infrapatellar fat pad [4]. The aetiology, clinical appearance, and diagnostic procedures of a ganglion cyst on the PCL are discussed in this case report.

## Case presentation

A 29-year-old male in training for a residency programme in orthopaedics surgery complained of pain in his right knee. He had never been in a traumatic situation. The discomfort developed as the training programme's mileage climbed. He had pain in his right knee while standing in an operating theatre for a long time. Running or walking distances of less than five kilometres were easily tolerated. After that, he would experience a sensation of fullness during knee motion. Brisk walking and lunges were the most common causes of pain, which would last for one to two days at a time. He had a pinpoint sharp pain on the most superomedial aspect of the patella that was relieved by gentle massage. Kneeling and full extension both caused pain. While moving the knee from flexion to extension, he experienced a brief intra-articular clicking sensation. Swimming and cycling were introduced to him as pain-free hobbies.

There was no oedema or bruises on physical examination. He had tenderness when the superomedial corner of the kneecap (which measured 1cm by 1cm in diameter) was palpated. From 90 degrees of flexion to full extension, the pain developed during the active range of motion. The McMurray's and Lachman's tests, as well as the anterior and posterior drawers test, were all negative.

**Figure 1:**
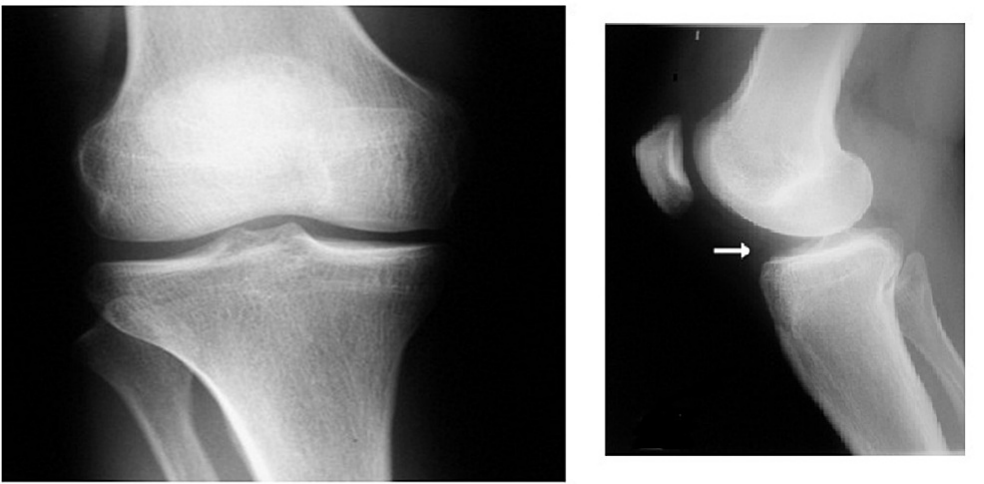
Plain radiography films of the knee in anteroposterior and lateral view showing no abnormality

**Figure 2:**
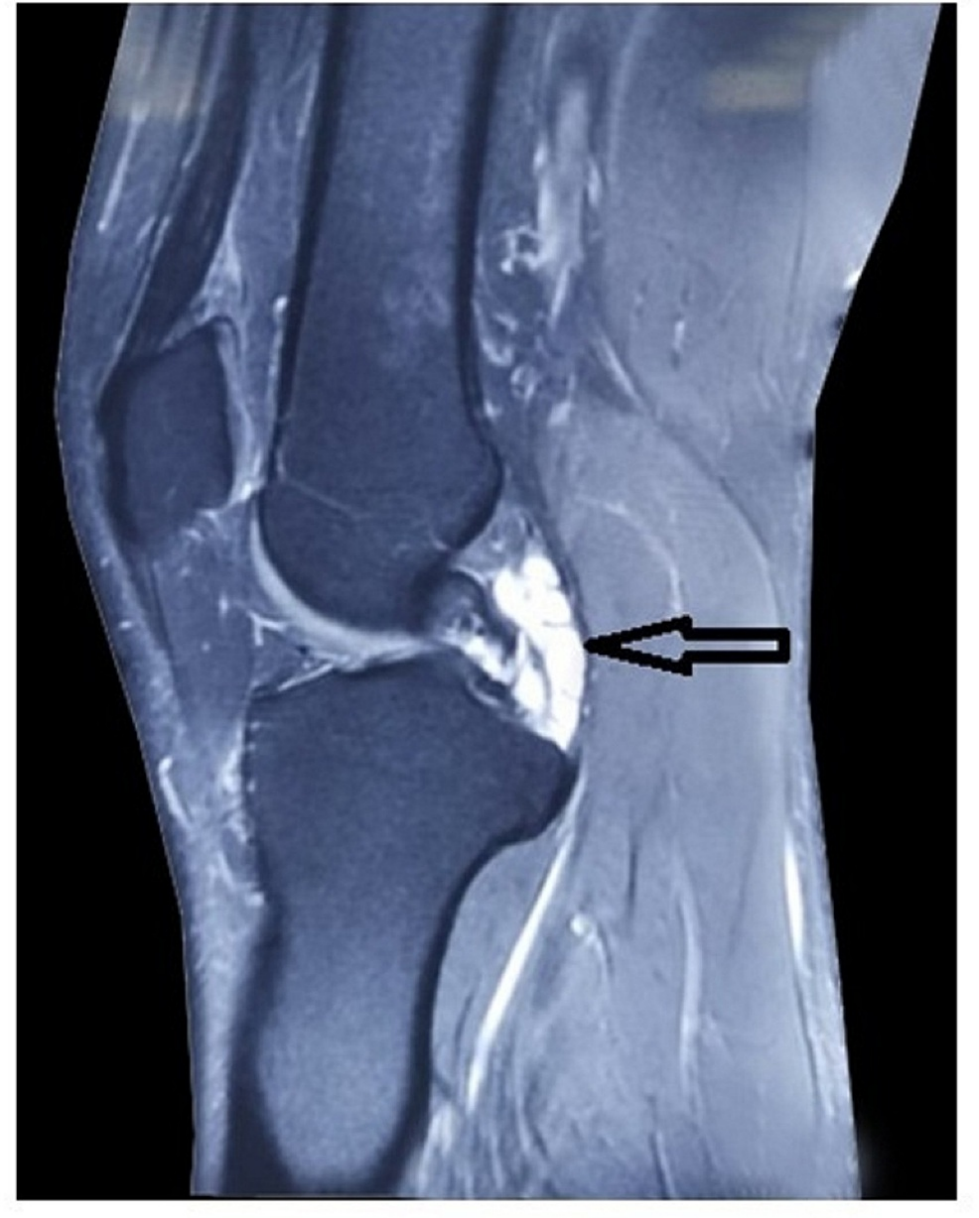
Saggital T2-weighted MRI showing a multiloculated cystic mass on PCL (black arrow)

**Figure 3:**
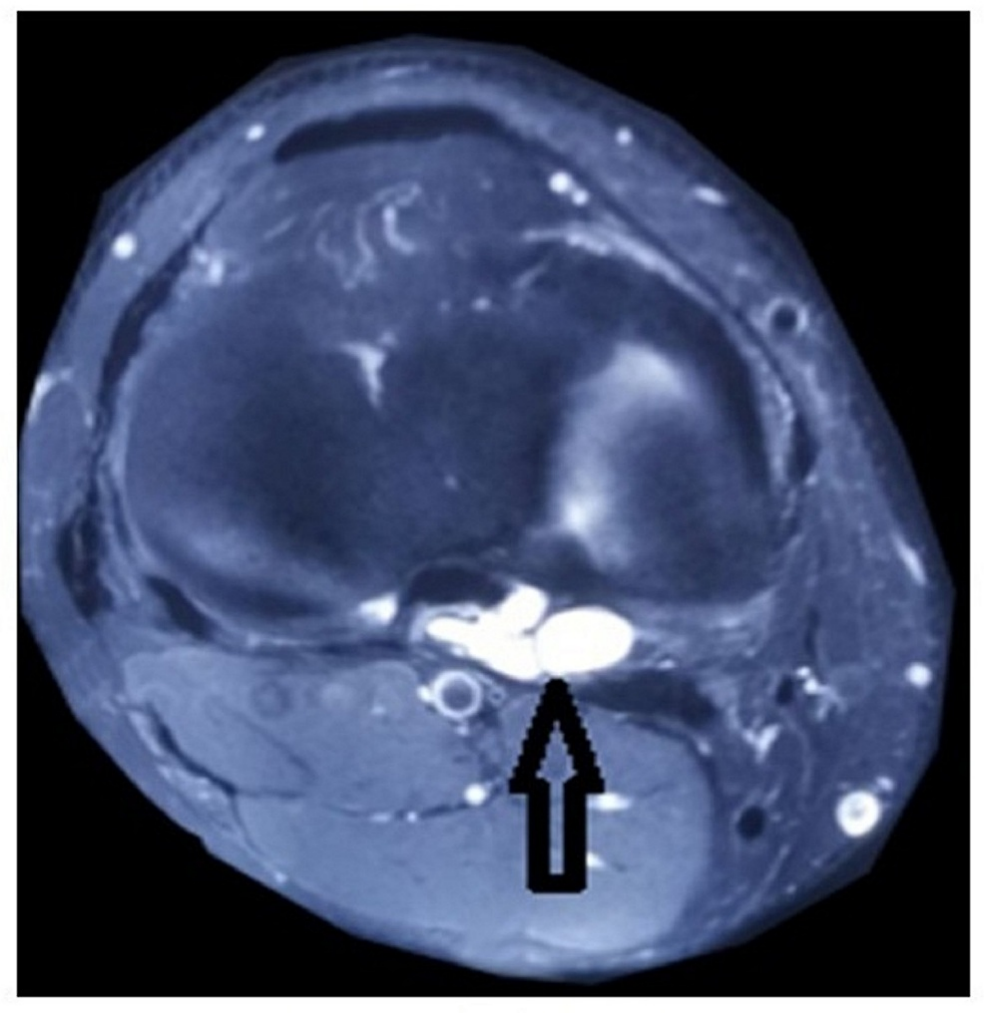
Coronal T2-weighted MRI showing multiloculated cystic mass on PCL (black arrow)

Plain film radiographs of the knee were taken; there was some blurring of Hoffa's fat pad's posterior boundary, but no abnormal bone structure or sagging body (Figure 1) was seen. Both knees were subjected to a diagnostic ultrasonography examination, which revealed no abnormalities. A multisequence MRI examination of the right knee revealed a modest weakening of the PCL ligament fibres at the medial condyle of the femur attachment site, as well as a multiloculated cystic lesion around the PCL (Figures 2-3). The patient was subjected to conservative management in the form of exercises like swimming, cycling and non-contact sports activities were encouraged surgical intervention was deferred due to the response shown by the patient.

## Discussion

Ganglions are cystic lesions with a clear, transparent, jelly-like fluid. They can form in menisci, muscles, and tendons [5,6]. Ganglia are generally found in locations that are subjected to constant mechanical stress [7]. Baker's cyst is the most prevalent type of peri-articular cyst in the knee, followed by synovial cysts and antefemoral [8]. Intra-articular ganglion cysts within the knee, on the other hand, are unusual but mostly grow in the cruciate ligaments' alar folds [9]. On MRI, the incidence ranged from 0.2% to 1.3%, and in arthroscopic studies, the incidence was 0.6% [2,3].

 An intraarticular ganglion can affect people of all ages, but it is more common in approximately 20-40 years of age and occurs most commonly in males [7,10]. Ganglia connected with the PCL are less prevalent than those associated with the ACL. Internal knee derangement frequently matches the clinical appearance of PCL ganglion [5]. They're tough to identify since there aren't many clear clinical signs and symptoms, so it takes a long time to figure out what's wrong. Ganglion cysts have an unknown cause. Cyst formation is attributed to mucoid degradation of collagen and connective tissues, according to the most frequently regarded physiological explanation [3-5].

A cyst is thought to originate as a result of trauma or tissue irritation, according to a more modern theory [3,5]. Synovial herniation, synovial tissue dislocation throughout embryogenesis, and proliferative pluripotent mesenchymal cells are some of the other possibilities for the aetiology of ganglion cysts. Although the specific aetiology of PCL ganglion cysts is unclear, mechanical stress is mostly on the posterior cruciate ligament while knee motion is considered to be the cause [11].

The PCL's ganglia are generally well defined, lobulated, and multiloculated on the ligament [11]. The mucin is transparent, highly viscous, and contains hyaluronic acid, albumin, globulin, and glucosamine. The ganglion is smooth-walled, translucent, and white [11]. In 87.5% of instances, the major cystic part originates posterior to a PCL, while in 12.5% of cases, it originates anterior to a PCL.

The clinical symptoms of PCL-related ganglion cysts are varied and non-specific [11]. Knee pain is the most common symptom, and it is frequently intermittent [3,10,11].

A stable knee will be revealed through an orthopaedic examination [6]. Functional activities including jogging, climbing stairs, and squats may aggravate the symptoms, which were the patient's chief complaints in this case. It's possible that the position, size, and dimension of a ganglion cyst are linked to the symptoms it causes, which can fluctuate over time [5]. Meniscal cysts, Meniscal tears, synovial chondromatosis, synovial proliferative problems, synovial hemangioma, as well as synovial sarcoma are among the clinical characteristics of a ganglion cyst on the PCL, which should be distinguished from internal derangement of the knee [12]. 

PCL ganglion cysts are usually discovered by chance while using diagnostic ultrasonography and MRI to rule out significant pathology. To rule out intra-articular loose bodies, plain film radiography is employed [6]. If this is negative, an MRI should be performed to see if there is any pathology [6]. 

MRI is the most sensitive, precise, specific, and non-invasive technique for demonstrating cystic masses, their size, and location. A magnetic resonance imaging (MRI) examination is also utilized to rule out cancerous tumours and discover other intra-articular diseases [4]. Anatomic, as well as morphologic linkages of synovial tissue to adjacent structures (e.g. bone, arteries, and soft tissues), can be identified using MRI [6]. Ganglia exhibit fluid features by having a low signal strength at T1 and a high signal intensity at T2 spin-echo or gradient-recalled-echo. A cyst's signal is normally homogeneous, but it can sometimes be heterogeneous, indicating the level of fibrous/myxoid transformation. The knee can also be evaluated with a CT scan or diagnostic ultrasonography. Intra-articular cysts present on CT as a well-defined water-density lesion and diagnostic ultrasonography as a hypoechoic cystic centre [13]. Special imaging is quite beneficial in rapidly diagnosing a ganglion cyst associated with a PCL.

Surgical methods are the most common treatments for PCL ganglion cysts. Arthroscopic resection is the most common and preferred method of treatment [2,4,7,9]. This is because it allows for the search for linked injuries, allows for thorough excision, has a lower recurrence rate, and usually leads to a quick recovery [3,4,9]. Nevertheless, arthroscopic resection is relatively costly, necessitates hospitalization, and may result in problems such as damage to the ligaments and popliteal arteries, as well as infection [9]. Cystic lesions have also been treated with CT and ultrasound-guided needle aspiration [3,7]. When compared to CT-guided aspiration, ultrasound-guided aspiration is rapid, easy to use, inexpensive, and does not release radiation [7]. However, when using CT or ultrasound-guided suction procedures, recurrence of cysts is possible in some cases.

The primary goal of this study is to educate clinicians about the diagnostic and treatment options for a posterior cruciate ligament ganglion cyst.

## Conclusions

To summarize, ganglia represent the result of inflammatory processes in the joint and are usually asymptomatic. In some cases, however, they can cause aesthetic discomfort, pain, or functional difficulties. The cyst wall is smooth and fibrous, similar to the synovial tissue that lines the joints and tendon sheaths. Most cysts do not require treatment, as they tend to regress spontaneously. However, if the ganglion is symptomatic, surgical aspiration or excision may be indicated. A diagnosis of ganglion should be kept while evaluating a painful knee with no obvious clinical findings. 

Surgical intervention is widely advocated but conservative management in the form of drugs and exercise can relieve patients with this condition to near normal activity levels. We advocate that physiotherapeutic modalities like ultrasonic exposure, fomentation, and changes in lifestyle involving exercising the knee can alleviate the symptoms leading to avoidance of surgical intervention. However, surgery should be considered if the patient has symptoms (pain and locking) after failed conservative treatment for six months and recurrence after aspiration of the cyst.
